# Is it time to rebalance the case mix? A portfolio analysis of direct catheterization laboratory costs over a 5-year period

**DOI:** 10.1186/s40001-016-0238-5

**Published:** 2016-11-03

**Authors:** Gunnar Plehn, Thomas Butz, Petra Maagh, Ahmet Oernek, Axel Meissner, Natalie Plehn

**Affiliations:** 1 Department of Cardiology, Johanniter-Hospital Duisburg Rheinhausen, Germany, Kreuzacker 1-7, 47228 Duisburg, Germany; 2 Ruhr-University of Bochum, Universitätsstrasse 150, 44801 Bochum, Germany; 3 Department of Cardiology, Catholic Hospital Oberhausen, Wilhelmstrasse 34, 46145 Oberhausen, Germany; 4 Department of Cardiology, Cologne-Merheim-Hospital, Ostmerheimer Strasse 200, 51109 Cologne, Germany; 5 Department of Radiology, University Hospital Bergmannsheil Bochum, Bürkle de la Camp-Platz 1, 44789 Bochum, Germany; 6 Schumpeter School of Business and Economics, University of Wuppertal, Gaussstrasse 20, 42119 Wuppertal, Germany

**Keywords:** Cardiac catheterization laboratory, Cost analysis, Contribution margin, Procedural portfolio analysis

## Abstract

**Background:**

Cardiac catheterization laboratories (CLL) have continued to function as profit centers for hospitals. Due to a high percentage of material and labor costs, they are natural targets for process improvement. Our study applied a contribution margin (CBM) concept to evaluate costs and cost dynamics over a 5-year period.

**Methods:**

We retrospectively analyzed all procedures performed at a tertiary heart center between 2007 and 2011. Total variable costs, including labor time, material, and maintenance-expenses, were allocated at a global as well as a procedural level. CBM and CBM ratios were calculated by integration of individual DRG revenues.

**Results:**

Annual case volume increased from 1288 to 1545. In parallel, overall profitability improved as indicated by a 2% increase in CBM ratio and a higher CBM generated per hour of CLL working time (4325 vs. 5892 €, *p* < 0.001). Coronary angiography generated higher average CBMs per hour than coronary or electrophysiological interventions (5831 vs. 3458 vs. 1495 €; *p* < 0.001). The latter are characterized by relatively high per case material expenditures. On a procedural level, DRG-specific trends as a steady improvement of examination time or an increase in material costs were detectable.

**Conclusions:**

The CBM concept allows a comprehensive analysis of CLL costs and cost dynamics. From a health service providers view, its range of application includes global profitability analysis, portfolio evaluation, and a detailed cost analysis of specific service lines. From a healthcare payers perspective, it may help to monitor hospital activities and to provide a solid data basis in cases where inappropriate developments are suspected. The calculation principle is simple which may increase user acceptance and thus the motivation of team members.

**Electronic supplementary material:**

The online version of this article (doi:10.1186/s40001-016-0238-5) contains supplementary material, which is available to authorized users.

## Background

Cardiac catheterization laboratories (CCLs) are modern and technologically advanced facilities offering specialized care to patients with a wide range of cardiac and vascular disease. They are frequently considered as profit centers within a hospital system [1]. Although the investments to create a CCL are high, hospitals were historically been able to achieve their economic return of investment rapidly because of the relatively high contribution margins (CBMs) on many procedures performed within the department [2]. During the last decade, realizing a return of this investment has become increasingly challenging. Hospital budget constraints, changes in DRG assignments, and a decreased level of public founding have put an enormous cost pressure on hospitals in many industrial countries [3–6]. In response, healthcare providers developed marketing strategies to increase patient number and throughput [2]. Furthermore, the importance of cost control instruments has increasingly been recognized [7].

Contribution margin analysis is the preferred financial analysis tool in situations where the profitability of different treatment types has to be compared within an established service line [8, 9]. Knowledge of the CBM is considered as an essential step to identify those procedures which contribute most to the coverage of hospital’s unavoidable fixed cost burden [10]. This applies particularly to the management of operating rooms or CCLs where time constraints are common. In such competitive bottleneck situations, expanded CBM analysis which also encompasses the CBM per unit of constraint time is preferably applied [11]. Since CBM analysis requires data on the individual patient level, it is more complex and time-consuming and was, therefore, infrequently used in the past [12].

The traditional costing systems do not separate between variable and fixed costs. As a consequence, time and material expenses are not appropriately allocated to specific procedures providing an unsafe base for strategic decision making. With the ongoing technical progress and the further development of hospital information systems, resource allocation at patient level will become widely available even paving the way for ad hoc analyses of individual procedure-related variable costs and CBMs [13].

With the beginning of the financial crisis in 2007, many health systems worldwide had to face with an increase in cost and competitive pressure [3]. Since cardiovascular disease represents a major economic burden in most countries, vigorous efforts to increase profitability were focused on cardiology units and in particular on cardiac catheterization laboratories [14, 15]. Based on our local experience, a significant increase in the number of treated patients and in total revenues has been realized in the meantime. However, an increase in case volume does not necessarily imply that economic performance improved as well. Based on a detailed analysis of material and labor expenses, our study provided a CBM analysis of typical CCL procedures over a 5-year period. Our objective was to demonstrate the excellent suitability of this approach to evaluate the economic performance of a catheterization laboratory on different hierarchical levels (overall and with respect to different service lines). Based on a detailed analysis of material and labor expenses, our study analyzed overall CCL profitability and the CBMs of typical procedure groups and procedures over a 5-year period.

## Methods

### Setting and data collection

Our study was performed in a hospital setting being reimbursed by diagnosis-related groups (DRG). The DRG system is the most widely used in-patient billing standard in Europe and follows a flat-rate per case approach. The German DRG system was adapted from the Australian DRG system and today classifies patients into about 1200 DRG groups according to their diagnoses and resource consumption [16, 17].

In total, 11,768 consecutive catheterization procedures from January 1, 2007 to December 31, 2011 of a University Medical Center in central Europe were analyzed. In a first step, patients’ baseline and procedural data were derived from the CCL database Metek (Metek, 52159 Roetgen, Germany) and the ongoing CCL patients’ registry. In particular, all materials utilized during the procedure were collected from the Metek database. The software provides a list of supply costs, including catheters and other disposable equipment, radiographic contrast medium, and medication. Each material position has been substituted in a synthesis step by a corresponding Euro amount, which has been generated from the purchase list of the material storage data base. In further step, the resulting database was combined with the hospitals’ information system (Clinicom CareCenter, Siemens). The corresponding DRG data were assigned to each case data set. As in most European countries, patients who had multiple CCL visits during hospital stay and such cases who received a combination of coronary and electrophysiological procedures during one visit were excluded. Furthermore, patients undergoing artificial respiration were excluded and those who received pacemaker therapy or any relevant procedure from other specialties (e.g., surgery or endoscopy). Patients with incomplete data sets were not included. All procedures were done by one of five interventional cardiologists with a high experience levels.

Subsequently, the three most represented service lines were defined: (a) coronary angiography (CA; *n* = 4632) which comprises all diagnostic left heart catheterization procedures; (b) percutaneous coronary intervention (PCI; *n* = 2187) representing all cases of coronary intervention; and (c) electrophysiological ablation (EA; *n* = 277) which defines all interventional cases in this sub-specialty. Furthermore, a selected cost analysis was performed on DRG level, including DRG F49E (*n* = 1810; rank 1) and DRG F58B (*n* = 373; rank 5).

### Contribution margin analysis

Our concept of CBM analysis implies the following: The assumed capacities in the area of in-patient care (e.g., room nursing costs) are considered as organizational prerequisite for the value creating process within the catheterization laboratory. These costs are accordingly added to structure or fixed costs. Costs, which are procedure-dependent generated and related to material, staff, cleaning, and maintenance are considered as variable costs and compared with the DRG proceeds. The CBM amounts were analyzed on a case-by-case basis. To take an account of time constraints, a CBM per hour of CCL activity was introduced. Furthermore, the CBM ratio was calculated which is the percentage of revenues which remain after all variable costs have been covered (CBM/DRG-revenue). Further details about the single methodic steps can be found in the specifications of the accounting model [6]. The study was approved by the Research Ethics Committee of the Ruhr-University of Bochum (register number 3945-11). All participants gave their consent to participate.

### Statistical methods

All data are given in terms of the mean ± SD. To compare group means with respect to systematic differences, an analysis of variance (ANOVA) has been performed. If variations between the means can be found, post hoc tests can be run to verify, which of both groups specifically differ. Before running post hoc analysis, the relevant factors were examined with respect to homogeneity (Levene-test). Depending on the results, either the Bonferroni test (variance homogeneity) or the Tamhane-T2 test (variance inhomogeneity) was performed. By means of a correlation analysis, it has been examined whether there is significance between two factors. Statistical analyses were performed using SPSS 16.0 for Windows (SPSS Inc. Chicago, IL, USA).

## Results

### Overall CCL performance

Analysis of the final data base revealed that the number of patients fulfilling inclusion criteria steadily increased during the observation period, rising from 1288 in 2007 to 1545 in 2011. At the same time, the average procedural examination time was substantially reduced from 0.77 to 0.61 h (Table 1). Since there was no significant rise in average DRG revenues between 2007 and 2011 (2842 ± 1615 vs. 2963 ± 1878 €; ns) and material expenditures (444 ± 591 vs. 476 ± 620 €; ns) remained unchanged, this improvement directly translated into a higher CBM per hour of CCL time (4325 ± 3937 vs. 5892 ± 5882 €; *p* < 0.001) and a 2% increase in CBM ratio (Table 1). General process parameters as the annual cumulative examination time (986 vs. 944 h) and the average duration of hospital stay (6.2 ± 5.9 vs. 5.8 ± 5.9; ns) did not change between 2007 and 2011.

**Table 1:** Cardiac procedure groups: economic data and their development

Procedural group	Year	*n*	CBM/h	CBM ratio	CBM/c	ET	ME	DHS
Coronary angiography	2007	821	4974 ± 4203	0.78 ± 0.20	1923 ± 1151	0.53 ± 0.34	227 ± 236	6.1 ± 5.6
	2008	836	5456 ± 5330	0.79 ± 0.15	1932 ± 1566	0.50 ± 0.41	210 ± 165	6.7 ± 6.3
	2009	963	5600 ± 4187	0.79 ± 0.17	1806 ± 1011	0.45 ± 0.36	218 ± 185	5.3 ± 4.6
	2010	1010	6303 ± 6116	0.79 ± 0.21	2042 ± 1608	0.46 ± 0.39	213 ± 172	5.9 ± 6.5
	2011	1002	6592 ± 5571	0.81 ± 0.19	2082 ± 1288	0.46 ± 0.41	204 ± 180	5.6 ± 5.4
	*F* test/*p*		14.7/<0.001	4.0/<0.001	6.4/<0.001	6.7/<0.001	1.9/ns	7.5/<0.001
	Post hoc 2007–2011/*p*		<0.001	0.002	ns	<0.001	ns	ns
Coronary intervention	2007	392	3409 ± 2953	0.68 ±0 .20	2469 ± 1859	0.96 ± 0.50	624 ± 522	6.1 ± 6.3
	2008	410	2826 ± 2526	0.62 ± 0.19	1988 ± 1137	0.97 ± 0.52	715 ± 409	5.7 ± 4.7
	2009	481	3232 ± 2785	0.60 ± 0.18	2059 ± 1341	0.84 ± 0.41	873 ± 486	5.0 ± 4.9
	2010	472	3376 ± 3213	0.60 ± 0.19	2141 ± 1854	0.83 ± 0.51	926 ± 527	5.1 ± 4.9
	2011	432	4444 ± 5617	0.65 ± 0.19	2565 ± 2341	0.79 ± 0.47	820 ± 531	5.3 ± 5.1
	*F* test/*p*		11.8/<0.001	14.3/<0.001	9.1/<0.001	11.7/<0.001	25.5/<0.001	3.5/0.007
	Post hoc 2007–2011/*p*		<0.001	ns	ns	<0.001	<0.001	ns
Electrophysiologic ablation	2007	56	1205 ± 2199	0.35 ± 0.22	2143 ± 1736	2.7 ± 1.1	2289 ± 949	7.3 ± 6.4
	2008	55	1625 ± 2610	0.39 ± 0.26	2169 ± 1653	2.3 ± 1.0	1978 ± 794	7.2 ± 4.5
	2009	50	1580 ± 3340	0.39 ± 0.21	2289 ± 1792	2.1 ± 0.83	2303 ± 999	5.9 ± 3.6
	2010	61	1461 ± 2192	0.37 ± 0.28	2176 ± 2079	2.2 ± 0.92	2223 ± 1237	6.1 ± 5.6
	2011	55	1623 ± 3999	0.36 ± 0.21	1708 ± 1057	1.8 ± 0.84	2423 ± 1331	6.4 ± 4.8
	*F* test/*p*		0.20/ns	0.26/ns	0.94/ns	6.6/<0.001	1.3/ns	0.78/ns
	Post hoc 2007–2011/*p*		ns	ns	ns	<0.001	ns	ns
Overall	2007	1288	4325 ± 3937	0.73 ± 0.22	2108 ± 1464	0.77 ± 0.65	444 ± 591	6.2 ± 5.9
	2008	1345	4490 ± 4727	0.72 ± 0.21	1998 ± 1491	0.73 ± 0.64	455 ± 507	6.5 ± 5.8
	2009	1534	4708 ± 3950	0.72 ± 0.20	1921 ± 1190	0.64 ± 0.53	495 ± 582	5.3 ± 4.7
	2010	1596	5219 ± 5512	0.72 ± 0.24	2100 ± 1757	0.65 ± 0.58	516 ± 629	5.8 ± 6.2
	2011	1545	5892 ± 5882	0.75 ± 0.22	2269 ± 1774	0.61 ± 0.56	462 ± 620	5.8 ± 5.9
	*F* test/*p*		24.5/<0.001	7.8/<0.001	10.8/<0.001	17.4/<0.001	3.9/0.004	9.5/<0.001
	Post hoc 2007–2011/*p*		<0.001	0.04	ns	<0.001	ns	ns

*CBM* contribution margin, *CBM/c* contribution margin per case, *CBM/h* contribution margin per hour, *DHS* duration of hospital stay, *ET* examination time, *ME* material expenditure

### Portfolio analysis

Coronary angiography was the most frequently performed procedure and generated the highest average CBMs per hour during the entire observation period [5831 ± 52905 € vs. 3458 ± 3631 € (PCI) vs. 1495 ± 2911 € (EA); *F* = 266; *p* < 0.001]. From 2007 to 2011, a steady increase in several profitability parameters as an increase in CBM per hour (4974 ± 4203 vs. 6592 ± 5571 €; *p* < 0.001) and CBM ratio (0.78 ± 0.2 vs. 0.81 ± 0.19; *p* < 0.001) was obvious. Examination time significantly decreased (0.53 ± 0.34 vs. 0.46 ± 0.41 h) and material expenditures remained unchanged. In contrast, an increase in CBM per hour values but not in CBM ratio was noted in coronary intervention procedures between 2007 and 2011 (Table 1). Although examination times improved significantly (0.96 ± 0.5 vs. 0.79 ± 0.47; *p* < 0.001), this increase in productivity did not translate into a favorable development of profitability in terms of CB ratio, since material costs significantly increased (624 ± 522 vs. 820 ± 531 €; *p* < 0.001).

Electrophysiological ablation was characterized by the lowest profitability from a CCLs perspective. Although CBMs per case were in a similar range as in the PCI and in CA group, CCL labor time was much more ineffective as indicated by low CBM per hour values. The reason behind the low profitability of EA procedures was high material expenditures (Table 1).

### DRG-specific cost analysis

Contribution margin analysis was applied in an exemplarily way to evaluate the development of DRG F49E (CA) and DRG F58B (PCI). Revenues of both DRGs increased within the observed time period. Simultaneously, the length of hospital stay and procedural examination time decreased. Shorter procedural examination times translated into higher CBM per hour amounts which indicated a higher profitability of CCL labor time. However, it is important to realize that these positive changes were paralleled by falling material costs in DRG F49E but steadily rising material costs in F58B. As a consequence of an increase in overall variables costs, CBM ratio of DRG F58B declined which indicates a gradual loss of profitability.

## Discussion

Our study, for the first time, reports on the application of a CBM approach to evaluate overall economic performance and to analyze the procedural portfolio of a CCL. Within the reported time period, a number of measures were implemented with a view to increase CCL performance. Examples include an improved scheduling of cases, a standardization of workflows, the introduction of flexible staffing, and a reduction in changeover times. Although total case number rose by 17% from 2007 to 2011, it was less clear if this increase resulted in a revenue surplus and thus a positive contribution to the economic performance of the hospital. In addition, there was uncertainty about the relative contribution of individual procedure groups to overall CCL performance. In the worst case scenario, the increase in case volume may have been attributed to a rapid accumulation of low or negative margin procedures and thus an unfavorable economic development.

To meet these uncertainties, we have in a first step applied the CBM analysis to evaluate overall CCL profitability on a year-by-year basis. It is important to recognize that an increase in CBMs per case may not necessarily indicate a better CCL performance, since there is a steady annual increase in DRG revenues which may per se contribute to higher per case margins. In our setting, however, a 2% increase in CBM ratio indicates that savings in CCL resource consumption and thus direct procedure-related costs were realized. Profitability per case rises as a larger share of DRG revenues contributes to the payment of hospitals’ fixed costs. Interestingly, these savings were solely realized by savings in examination time, since material expenses hardly changed over time. Time management has previously been identified as an important target for CCL process improvement [14, 18]. Cohen et al. analyzed aggregated data of 70 catheterization laboratories and revealed a high variability of CCL examination time. In particular, high-volume centers were found to realize time savings of 5–9 min per procedure. Since cardiac catheterization requires special technical skills and training, it seems obvious to assume that physicians’ experience is a major determinant of CCL resource consumption. However, a cost analysis of 250 PCI cases revealed no correlation between physicians experience and PCI costs, although physicians accounted for 19% of the variation of hospital costs in this study. In addition, there was no difference in procedural success rate among physicians performing high- and low-cost procedures. It was, therefore, concluded that different workflows may largely contribute to the variation in CCL resource consumption and that these practice habits provide a high potential for process improvement [19]. Our findings confirm this assumption in demonstrating that examination time is a highly modifiable determinant of procedural costs and that a significant improvement can be achieved within an established team and experience level. It was also shown that time savings can be effectively used to realize volume growth which, in turn, generates additional revenues and CBMs. CCL capacity is limited in many countries and typically represents the bottleneck of patient flow in cardiology [4, 5]. Contribution margin analysis is a useful tool to determine the effect of management changes in this setting. Its application helps to identify if strategies to improve the capacity of the entire system are effective. Therefore, it is hardly surprising that the CBM approach has successfully been applied in medical areas where severe time constraints are common as emergency departments and acute care surgery divisions [8, 11].

**Fig. 1:**
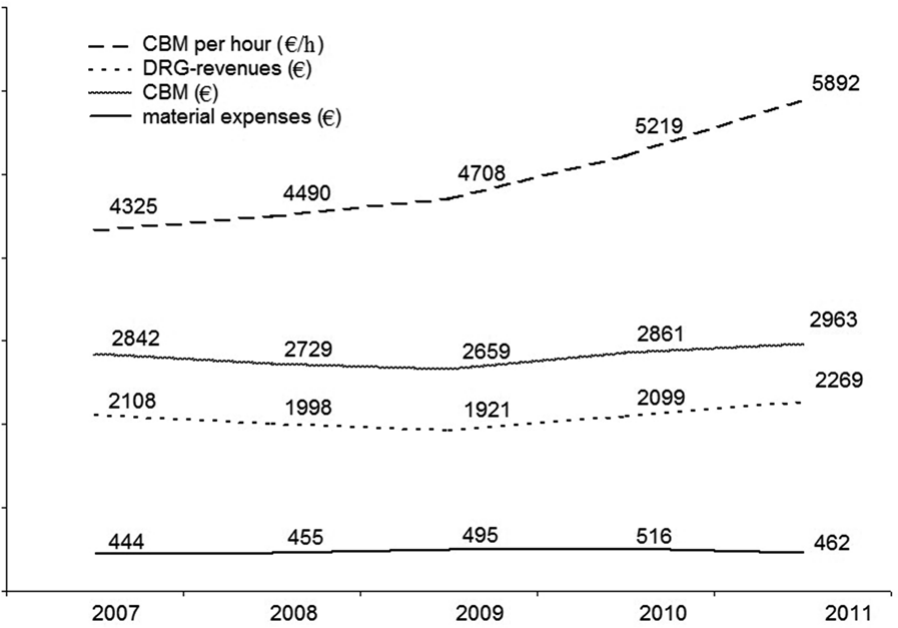
Annual average performance data per case

From a hospital’s economic point of view, it is not only important to determine overall profitability but also to figure out how profitable different service lines are. The CBM concept was, therefore, applied in a second step to compare typical CCL service lines and to identify those that give the highest returns. Among the three procedure groups analyzed, CA proved to generate the highest CBMs per hour and was performed with the highest case frequency per year (Fig. 1). In addition, its case volume increased during the study period. Theoretically, this expansion represents the best-possible economic variant. In a “bottleneck” situation, any spare capacities available would be most profitable used by the service which generates the highest CBM per hour. Accordingly, 1 h of PCI or EA service would perform worse than 1 h provided for CA. However, it must be emphasized that three service lines generated positive CBMs and, therefore, contributed to the covering of hospitals fixed costs. The relatively low CBMs gained from EA procedures were unexpected, since these procedures are performed in an expensive electrophysiology laboratory usually involving numerous physicians. A study of Winkle et al. pointed out that there is a wide range of expensive equipment which can be used for atrial fibrillation ablation. Depending on the physicians choice, a threefold range of material costs was observed. However, there was much concern that even the lowest-equipment price scenario was adequately reimbursed by Medicare DRGs [20]. We suppose that similar mechanisms may explain the relatively low margin of EAs in our study. To reduce variable costs, it is, therefore, important to carefully choose and negotiate catheter equipment in this field.

Although portfolio analysis is a proven strategy in business and industry, such concepts have only recently entered healthcare industry. On the one hand, it is important to recognize that the majority of hospitals have to fulfill public service obligation and cannot arbitrarily cease unprofitable service lines. On the other hand, information on low- and high-margin services can well be used to further develop or rebalance the case mix. If low or negative CBMs are generated, the variable costs structure of these service lines has to be questioned critically to reduce these expenditures. In addition, other options as hospital co-operations or out-patient service models may be considered to improve process efficiency. Identifying high-margin service lines may help to focus development strategies, marketing efforts, and investments into areas of future growth with the aim to increase the volume of these cases. A study on the implementation of portfolio planning in plastic surgery confirmed that such efforts are effective in improving hospital’s financial position and patients’ satisfaction [21, 22] (Additional file 1).

**Fig. 2:**
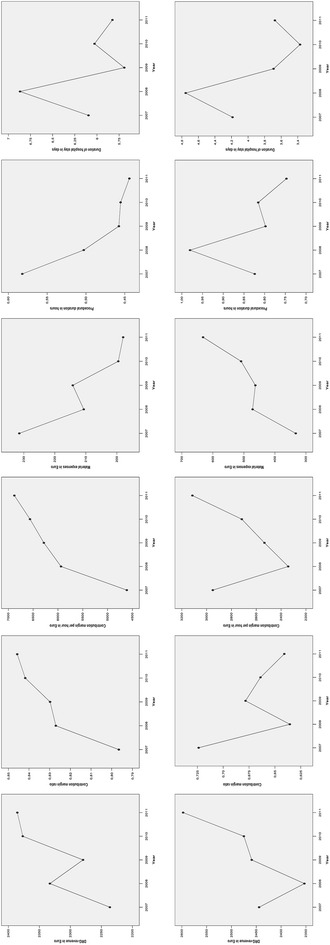
Performance pattern of DRG F49E (ranking number 1) and DRG F58B

Third, we demonstrated that CBM analysis when converted to a case-based measure provides valuable insights into the mechanisms underlying positive or negative economic developments. The cost structure of a CCL is characterized by a high percentage of manual activity and high material expenses. Given the high proportion of variable costs, even small reductions in these expenditures could have a direct positive impact on revenue surplus as CBM will increase. In the case of DRG F49E, savings in material costs and in labor time had a synergistic effect on CBM development. A divergent behavior was obvious in DRG F58B cases. The efforts to improve time management were almost used up by a steady increase in material costs. This example makes us aware how important it is to identify relevant cost drivers and their dynamics to develop appropriate strategies to support positive and to counteract negative trends (Fig. 2).

Within a DRG-based payment system variations in service line profitability may create incentives to invest in profitable lines over less profitable ones which, in turn, may lead to the provision of inappropriate services. Such incentives in hospital activities are inherent to a per case reimbursement mechanism and are, therefore, not completely avoidable. To overcome such unintended consequences, basic models of DRG-payment are typically modified by regulatory instruments. In Germany and other European countries, provider-level budgets or ceiling was introduced to counterbalance incentives to treat more cases. More important, data on coronary angiographies and interventions are integrated into federal or national quality assurance programs. Recent reports of these institutions ensured that the indication of these procedures were in line with the current guidelines and not driven by economic aspects [23].

With respect to the ongoing development of the German DRG system, the observed imbalance in the profitability of different service lines is not expected to disappear in the near future. When typical interventional or EA procedures from 2011 were virtually compared with their 2015 equivalents, little increases or even decreases in total DRG revenues can be noted [24]. Double digit increases in DRG revenues were exclusively reserved to cases with diagnostic cardiac catheterization. One can, therefore, assume that the observed gap between diagnostic and therapeutic procedures may even become wider. This issue points at an important limitation of the German DRG system. Resource consumptions in terms of material costs and time input are poorly reflected by the current German DRG version, and from an European perspective, most national DRG systems were found to perform better [25, 26]. Placing our findings into this context may help to refine the current DRG algorithms.

The study represents a single center experience and generalizability may thus be limited. Processes show a considerable variation across cardiac catheterization laboratories and material costs may differ depending on hospital contracts. However, institutional characteristics do not interfere with the transferability of our CBM concept but may indeed encourage its application. With the ongoing evolution of hospital information systems, even ad hoc analyses are conceivable.

The CBM concept does not include fixed costs, such as room nursing costs, laboratory costs, and overhead costs. However, those costs (variable costs) which can be directly influenced by the catheterization team are well reflected.

## Conclusions

The CBM concept allows a comprehensive analysis of costs and cost dynamics in the setting of a catheterization laboratory. Its range of application includes global profitability analysis, portfolio evaluation, and a detailed cost analysis of specific service lines. The calculation principle is simple and the concept solely includes cost components which can be influenced by the catheterization team. This fact may increase user acceptance and thus the motivation of team members. Contribution margin analysis can be viewed as an integrative tool which can manage costs and the value creating process together.

## Additional file

**Additional file 1.** Excel sheet of the original data base.

## Abbreviations

CA: coronary angiography
EA: electrophysiological ablation
CCL: cardiac catheterization laboratory
CBM: contribution margin
DRG: diagnosis-related groups
PCI: percutaneous coronary intervention
